# Using the internet for suicide-related purposes: Contrasting findings from young people in the community and self-harm patients admitted to hospital

**DOI:** 10.1371/journal.pone.0197712

**Published:** 2018-05-24

**Authors:** Lucy Biddle, Jane Derges, Carlie Goldsmith, Jenny L. Donovan, David Gunnell

**Affiliations:** 1 Population Health Sciences, Bristol Medical School, University of Bristol, Bristol, United Kingdom; 2 Samaritans, Ewell, Surrey, United Kingdom; 3 NIHR CLAHRC West (National Institute for Health Research Collaboration for Leadership in Applied Health Behaviour and Care West), University Hospitals Bristol NHS Trust, Bristol, United Kingdom; 4 National Institute for Health Research Bristol Biomedical Research Centre, University Hospitals Bristol NHS Foundation Trust and University of Bristol, Bristol, United Kingdom; University of Queensland, AUSTRALIA

## Abstract

Despite accelerating interest in the impact of the internet on suicidal behaviour, empirical work has not captured detailed narratives from those who engaged in suicide-related internet use. This study explored the suicide-related online behaviour of two contrasting samples of distressed users, focusing on their purpose, methods and the main content viewed. In-depth interviews were conducted in the UK between 2014–2016 with i) young people in the community; and ii) self-harm patients presenting to hospital emergency departments. Data were analysed using methods of constant comparison. Suicide-related internet use varied according to the severity of suicidal feelings. In the young people sample, where severity was lower, use was characterised by disorganised browsing without clear purpose. A range of content was ‘stumbled upon’ including information about suicide methods. They also pursued opportunities to interact with others and explore online help. Self-harm patients were a higher severity group with a history of suicidal behaviour. Their use was purposeful and strategic, focused around ‘researching’ suicide methods to maximise effectiveness. They made specific choices about content viewed; many consulting factual content in preference to user generated accounts, while help content and communication was avoided. Findings indicate further action is necessary to improve online safety. Also, novel online help approaches are needed to engage individuals experiencing suicidal crisis. Awareness of the nature of suicide-related internet use and how this may reflect the status of an individual’s suicidal thinking could be beneficial to clinicians to promote safety and indicate risk.

## Introduction

The internet and social media have been recognised as of great importance to suicide and suicide prevention[1]. Internet search studies confirm that pro-suicide discourse and technical information about suicide methods is highly accessible[2, 3], significant relationships have been found between suicide-related search trends and rates of suicide[4], and online information may increase awareness of high-lethality methods[5]. The risks posed by the online world are evidenced through numerous case studies of ‘cybersuicide’ where the individual’s suicide is reported to have been facilitated through information or encouragement obtained online (e.g.[6–8]). Counterbalancing this, there has been considerable discussion about the internet’s potential to deliver suicide prevention through support sites and forums[9, 10], links to help services[11] and online mental health interventions and apps[12].

A recent study found nearly a quarter (22.5%) of young adults are exposed to online information about suicide and self-harm, and 9% have used discussed suicidal feelings or self-harm online[13]. The 2017 National Confidential Inquiry into Suicide and Homicide[14] found suicide-related internet use in 26% of deaths in those under 20 years and 13% of deaths in 20–24 year olds. In a survey of adults presenting to an Emergency Department (ED) following self-harm, 9% reported suicide-related internet use in relation to the episode[15].

Despite a burgeoning research literature, full understanding of the interplay between suicide-related internet use and suicidal behaviour remains unclear. Research exploring the internet’s potential as a medium for suicide prevention tends to be descriptive rather than evaluative[10, 16], and the conclusions that can be drawn from prevalence estimates, internet search studies and other quantitative approaches are limited by a lack of direct engagement with users[4]. Existing user research is typically based on individuals responding to online surveys (e.g.[17–19])

We conducted a large-scale study exploring suicide-related internet use and its impact on suicidal behaviour. This paper describes findings from two qualitative samples and reports on the purposes and methods of suicide-related use, including the types of content viewed.

## Methods

In-depth interviews were conducted between 2014 and 2016 with two contrasting samples: i) young people in the community aged 21–23 years, drawn from The Avon Longitudinal Study of Parents and Children (ALSPAC)[20]; ii) patients presenting to Hospital EDs following serious self-harm. The research was approved by the Frenchay Research Ethics Committee and the Ethics and Law committee for ALSPAC.

### Young people sample

ALSPAC is a population based birth cohort examining influences on health across the life-course[20]. Questions on suicide-related internet use and history of self-harm were included in a questionnaire sent to 8525 participants when they were aged 21years[13]. 48.2% responded and 46.3% provided data on internet use. Suicide-related internet use was defined as coming across or searching for information on self-harm or suicide (including help), or using the internet to discuss self-harm or suicidal feelings with others. From those reporting suicide-related internet use (22.5%, n = 3946), purposive sampling was used to select males and females reporting varying severities of suicidal feelings and behaviour, including: a main group who had self-harmed in the previous year with suicidal intent; and a small subgroup who reported internet use and either suicidal feelings but no behaviour, or only self-harm without suicidal intent. In total, 273 eligible participants were contacted of whom 30 expressed an interest in participation and 13 proceeded to interview. The sample included individuals who had not attended services (n = 3). Participants were recruited approximately 18 months after questionnaire completion. Some participants remained active or occasional users at the time of interview, while others were retrospective cases, whose suicide-related internet use had since resolved, and they reflected on this past use.

### Self-harm patients sample

Participants were individuals who presented to the EDs of two acute hospitals in South West England following self-harm, and who reported any suicide-related internet use in relation to the episode during psychosocial assessment with Liaison Psychiatry. Typically, such patients represent a moderate to high risk group who present with suicidal intent. The mean Beck Suicide Intent Scale (SIS)[21] score for patients assessed in 2015, when participants were recruited, was 8 (indicating moderate intent[22]). A proforma including a question about suicide-related internet use is used in the assessment. Individuals were recruited prospectively by assessing clinicians over a period of 18 months, and retrospectively (past year) following a search of the assessment proformas. Clinicians invited patients they deemed well enough to take part and with capacity to give informed consent. Sixty-five individuals were invited and 20 participated.

In each sample, recruitment continued until a range of participants had been interviewed, including some negative cases, and consistent findings were emerging.

### Data collection

Interviews for each sample were conducted by JDe using the same protocols and topic guides as far as possible. JDe was an experienced senior researcher with a background in mental health research. She had no prior relationship with participants. Most interviews took place face-to-face at a location chosen by the participant. One was conducted by telephone. All were audio-recorded and took place with the participants’ prior written informed consent. Data collection and analysis occurred simultaneously according to principles of grounded theory[23], interviews being conducted in batches with parallel analysis.

An interpretative approach was adopted where participants were encouraged to talk in depth, identifying the issues they considered to be of importance. This made it possible to obtain detailed narratives of individuals’ internet use and the meanings it held for them in relation to their suicidal thoughts and behaviour. A topic guide (S1 Text) ensured core issues central to the research question were explored with each participant but this was used flexibly according to the direction taken by participants and underwent several iterations, being revised in response to emerging themes so these could be explored further. Probing was used to clarify salient points and encourage reflection. Interviews explored all internet use where suicide was the focus of content or dialogue. This was explored in relation to specific episodes of suicidal behaviour and generally throughout the individual’s narrative where suicidal intent may or not may have been present. With few exceptions, most interviews lasted between one and two hours. A small number exceeded two hours.

### Analysis

Interviews were transcribed verbatim. The two samples were analysed separately, but following the same procedures, each led by a different researcher (JDe: young people; LB: self-harm patients). Transcripts were coded to identify key themes and ideas. Approximately a third from each sample were independently double coded by the other researcher to check reliability and reach an agreed coding frame that could be applied consistently to all transcripts from the sample. Codes were amalgamated into higher order concepts, or sub-divided as a more refined understanding emerged. To aid comparison, attempts were made to assimilate the coding frames of the two samples where possible and to use consistent terminology, but primacy was given to ensuring the data grounded the coding frame for each sample. Analysis progressed using the methods of constant comparison[23]. Data relating to each code were examined closely for content and compared within and across individuals to identify similarities and differences and how these could be accounted for. This included a focus on ‘deviant cases’, contrasting helpful and harmful internet usage, and exploring the sequencing and advancement of themes over time in longitudinal narratives, and according to levels of suicidal intent. Descriptive accounts of major themes were produced. NVivo v. 10 (QSR International) was used to assist analysis.

## Results

Thirteen young people from the community sample (hereafter ‘young people’ (YP)), and twenty hospital-presenting self-harm patients (hereafter ‘self-harm patients’ (SH)) were interviewed (Table 1). Most of the young people were 23 years at the time of interview. Self-harm patients represented a range of ages, though a third (35%) were ≥24 years. Similar numbers of men and women were recruited, and individuals with varying employment and marital status. Psychiatric diagnosis (including depression and anxiety) were common in both groups, but self-harm patients reported more severe suicidal behaviour and diagnoses including borderline personality and bipolar disorder. While most of the young people described suicidal thinking and over half had self-harmed, only three had attempted suicide, compared to all except one of the self-harm patients, many of whom had made multiple attempts. Most of those attempting suicide had overdosed, though some also described episodes of attempted poisoning, hanging and jumping. Three of the young people had presented to hospital following self-harm and while most had received intervention from health or education services, three reported no service use. Self-harm patients reported high levels of medical service use in addition to their hospital attendances.

**Table 1:** Participant characteristics.

Characteristic	Community-based young people sample	Self-harm patients presenting to ED
**Age**		
Range	22–24 years	19–51 years
Mean	23.0 years	31.6 years
**Sex**		
Male	6	8
Female	7	12
**Current marital status**		
Single	12	12
Married/ lives with partner	1	2
Divorced/ separated	-	5
Widowed	-	1
**Employment**		
Employed/ self-employed	4	9
Employed p/t	4	-
Student	5	4
Unemployed	-	5
Looking after child	-	2
**Psychiatric diagnosis**		
Yes	8	15
No	5	5
**Suicide attempts**		
Unclear	2	-
None	8	1
1–3	3	11
More than 3	0	8
**Service use**		
Professional medical	9	17
Emergency Department	3	20
Counselling/ therapy	5	9
Voluntary sector	1	2
None	3	-

Participants accessed the internet using various devices including laptops, desktop computers and phones. All described some suicide-related use and many described multiple episodes, presenting extensive data for analysis. The samples displayed differing internet behaviour in relation to key themes, as detailed below.

### The purpose of suicide-related internet use

Both samples viewed a broad range of online content about suicide and suicide methods. Amongst the young people, this was mostly gathered with mixed, unclear or changeable purpose, reflecting the fluctuating moods and ambivalence about suicide characterizing this group. Essentially, these participants looked online to try to make sense of their suicidal feelings and behaviour, which involved them exploring suicide as an idea while also looking for opportunities to obtain help or support (below). They sought explanation, reassurance, ways of coping, and possible solutions, including suicide. Some described their activity as motivated largely by ‘interest’ (YP1,13), ‘intrigue’ (YP8), or ‘curiosity’ (YP10). Use increased and became more focused on negative content when mood lowered, and decreased where mood improved. Their most commonly articulated purpose was a desire to read the stories and views of others who felt suicidal or engaged in suicidal behaviour. They hoped to find others they could relate to, or compare themselves with, to understand their own situation.

I could just read [on self-harm/ suicidal behaviour chatroom] what everyone else was saying. I was really interested in the way that others were talking about it and it would be like a nice little comfort blanket… it was literally just like not feeling so alone (YP9)

I did used to search up stories of suicide… I don’t actually know why. I think I wanted to relate to people… so I felt like it wasn’t just me. So I’d go online and search up suicide and stuff and I’d search up ‘girl commits suicide UK’ and I would just read loads of articles about girls and boys who had done it. All their various reasons (YP3)

In contrast, three-quarters of self-harm patients developed a specific purpose to their suicide-related activities, focused on investigating suicide methods in the context of planning, or intending to plan, suicidal behaviour. They described this as ‘researching’, some distinguishing it from the casual browsing which typified the young people:

It’s [use] definitely getting that idea in my head that ‘oh I just can’t be bothered with life anymore. Oh, I’ve got an idea [possible method], let’s go and research it’. That’s when you’re sort of purposeful rather than just randomly thinking ‘oh, I’ll just research sort of little things’ (SH20).

Some described episodes of unfocused use reminiscent of the young people. This was common earlier in their suicidal narrative or between acute episodes where suicidal feelings were milder. Use became purposeful as suicidal intent increased:

The more time you spend alone and depressed, the more time you overthink your depression… the internet’s always in your hands so I would just every now and again just look into [suicide]… it was intent but a lot of the time it was so casual. It was like when you’ve got a really good job but you still have the odd look at jobs on the market… (Int: Around the time you became more focused on making your attempt, did anything change about your search behaviour?) I was more specific ‘cos I knew the drugs I had at home (SH14)

Around half the young people viewed content about suicide methods, but with just two exceptions this was at a superficial level. This compared to the self-harm patients whose use was often extensive, with many examples of this having shaped a subsequent suicide attempt; the depth and immediacy of their research corresponded with levels of suicidal intent.

Specific purposes of use amongst self-harm patients are illustrated in Table 2. Nearly all went online to obtain further information about methods they knew or theorized about. Half researched the usability of items within their immediate environment, including over the counter and prescription medicines and household chemicals. Most also sought new or recommended methods ranging from identifying ‘least painful’ or ‘most effective’ methods, to searching content for the novel or ‘exotic’ (SH2). Their research often followed a prior suicide attempt uninformed by internet research and was motivated by wanting to now avoid ‘failure’ (SH5) and secure a more ‘effective’ (SH8), ‘clever’ (SH1), or ‘scientific’ (SH2) attempt. They therefore researched implementation in some depth, seeking technical instructions to help them plan. Examples included looking up fatal drug doses, jumping heights, ligature points, noose tying, preparing poisons, and timescales ‘before the point of return’ (SH2). They also searched for, and in six cases purchased, items for suicide online, including syringes, prescription-only drugs, and poisons. Most self-harm patients sought content which enabled them to compare and evaluate methods. This spanned from ratings of speed, pain, certainty, ease and ‘goriness’ (often contained within lists and tables of methods displayed on suicide sites), to information about possible outcomes of failure and the subjective experiences of those who had attempted. Such information was used to assist with decision-making and could lead to individuals being deterred from methods if they appeared risky or unpleasant.

**Table 2:** Purposes of suicide-related internet use amongst self-harm patient participants.

Purpose	Data extract
***Researching known methods***	Something in my head clicked that I know people have accidental deaths because may be they have a gas fire that isn’t working properly and they can simply feel tired, fall asleep and never wake up. Um, and so I thought I would turn again to the internet for research on that (SH20)
***Researching everyday items***	My Mum takes loads of different medication. She’s got a basket like that and I went through it one night and there’s one in there called [name of drug] and I looked up that online and that seemed quite like it would work (SH1)
***Seeking new/ recommended methods***	I’d overdosed on prescription drugs, my anti-depressants, that hadn’t worked… I tried paracetamol, that didn’t work. So, I thought I’d go on the internet and look up the ten worst poisons there are and I started investigating poisons (SH5).
***Seek details of effective implementation***	I took [ligature] and thought, ‘I’ll do it with this”…so then I Googled, ‘how to hang yourself’… it was an in-depth report on how Saddam Hussein had been hanged and the knot had to go [states location] to break your neck (SH5)
	I started looking up dosages and if I was going to introduce alcohol, how much alcohol, because it is certainly a great catalyst for everything, but you know, too much then you’re going to be vomiting it all up… and yeah whether a cocktail was best and a whether a lower dosage or a staggered dosage–that sort of thing… it was a lot more regulated how I was handling it rather than just chugging them all back (SH2)
***Seek evaluation of methods***	I read through the options [in suicide lists] … it was like, ‘what can I do that means within the shortest amount of time, I’m gone’, so yeah, I did, I went through all of them (SH15)
	I typed in ‘how to hang yourself’… I thought it would be quick, painless and no mess really… I didn’t do it because hanging is about breaking your neck and if you don’t do it properly its asphyxiation …and I was scared I’d do it wrong (SH14)
	I’d get an idea in my head of ‘right I could do this, that would be a good way to die. I’ll just investigate that–see if there’s anything online to sort of say how it would feel, how it would work, how easy it is, is it going to be a good option or is it something where there’s going to be a lot of suffering?’ (SH20)

Comparable activity only occurred amongst the young people when they were in acute distress. A small group looked up specific methods, exploring characteristics such as certainty and painfulness, but they rarely researched in depth and their purpose remained largely speculative or hypothetical. One exception researched a novel method with a view to possible implementation. She contrasted this with her previous suicide-related internet activity, attributing the change to her heightened suicidal intent.

That’s when I went on a forum ‘cos I wanted to look up a way, because I know how I’d been in the past–I’d never quite like (pause) I kind of just teased the idea but at this point mentally I was quite committed to die but I wanted to find a way to do it. I didn’t want to die slowly. I didn’t want to die in pain… I started really searching other ways… I remember being quite set on this kit you could buy… [describes method]… I pasted that in a new Google search to see what would come up, whether it would come up with reports of people who had died, which it did (YP10).

### Internet search behaviour and content viewed

Participants in both samples were high frequency internet users, though this was most evident amongst the young people who had an almost constant presence online. Their suicide-related use was often an extension of everyday use and characterised by unstructured browsing of a broad range of content. Entering the search term ‘suicide’ (or a variant of this) and viewing ‘what came up’ (YP1) rather than looking for specific material was a typical and repeated activity:

Every day I would Google ‘suicide’, just as a thing. I guess it was a bit of an obsession … and I would just go through [hits] seeing what was going on. Well some of them were positive… and some of them were about people who had taken their lives or attempted… (Int: what was your intention at that point?) I don’t know to be honest… I would have seen both and looked at both (YP13)

Another common search strategy amongst the young people was impromptu entering of questions or statements about suicide or one’s feelings (e.g. ‘how painful is it to kill yourself?’; ‘I’m too sad to live’; ‘I want to kill myself’; ‘I want to die’). This could yield pro-suicide and prevention content. Around half the participants entered search terms such as ‘stories of suicide’, ‘suicide blog’, or more directive searches around suicide methods—though (other than in a few episodes of acute distress) these were mainly confined to broad terms such as ‘successful suicide methods’.

In contrast, only four self-harm patients reported entering the generic search term ‘suicide’. Some described initially using non-specific search terms while scoping methods, or when feelings were milder, but typically they described an accelerating trajectory of online research where their searching became more specific and strategic. Early searches informed the next and specific terms were used to target chosen methods.

I started to get kind of suicidal ideation and it [internet use] more started out as a hypothetical thing… just, ‘if I was going to do [suicide], how would I do it’… as it went on I was more and more Googling stuff… and then I got really, really depressed and that’s when my research got very specific. So instead of Googling ‘easiest method of suicide’, I was Googling stuff like the drug used in lethal injections (SH6)

While self-harm patients selected from search output, young people navigated output with little discrimination, most clicking on the first hits and then working through, or randomly according to which piqued their interest at that time:

I used to go to [Ask Jeeves] and find what would come up and I used to go on different ones… ‘cos there’s loads, there’s hundreds… so I tended to look at everything that there was (YP8)

In fact, a disorganized trail through suicide content was common in the young people sample. They followed links within and across sites, on newsfeeds and social media, clicking on ‘anything that came up’ (YP6), such that they described ‘stumbling upon’ (YP8) material, often with ‘no idea how they got there’ (YP6), and unable to recall site names. This tendency increased as mood lowered, and escalated browsing, causing them to deviate considerably from their starting point and to explore issues they had not considered. They encountered content describing or depicting methods of suicide during such unfocussed browsing:

I unconsciously found myself using [Q&A site] because it’s so big. So many questions are asked on there and I guess I used it more than I thought because they put the links at the bottom to related questions… and you could see the stages [of suicidal feelings] progress and how the responses progressed (YP4)

When you’re Googling stuff on the internet and then you’ll go down another route and then another route and then you start following links and sort of read something and stop at something and then open a new tab and look into that and, yeah, just learning different ways to die (YP10)

Many of the young people were exposed to graphic content, which could impact upon their ideas, either suggesting methods or revealing them to be unpleasant:

That [site] led me to a link about the Golden Gate Bridge and then I watched a film called, ‘The Bridge’, which was outstanding. Um, I watched that before I was actually debating it. I don’t know if it was subconsciously fueling it but that was how I planned to go… I never consciously sought out to watch… but to watch that and see, because they set up the cameras, didn’t they? And they show you the people falling (YP4).

So, websites that show really graphic images… there was one that was how to shoot yourself properly and it was horrendous because there was loads of pictures of people who had failed… (Int: can you remember the name of that site?)… I can’t remember because you just scroll through and you get up a website and then you get onto another one… (YP6)

Most young people browsed large quantities of material including public opinion about suicide, ‘pro-suicide’ views, experiential accounts and online dialogue of others who had felt suicidal or been bereaved by suicide, and information about suicide methods. Key sources were ‘Baww threads’, video stories, news reports, suicide sites, forums, chatrooms, and ‘question and answer’ sites.

There were fewer references to ‘stumbling across’ content amongst the self-harm patients. While some had initially viewed a wide range of material, departing from the young people, they made increasingly deliberate choices about which types of content to select for further viewing and which would yield ‘better answers’ (SH10). A common strategy was to search for case studies of suicide or suicide attempts. Examples included: publicized suicides, documentaries, posts of individuals who had attempted suicide; clinical case-studies; and reports of non-suicide deaths from the same means. Completed suicides were studied as ‘successful’ examples that could be replicated.

It was around the time that Mick Jagger’s girlfriend took her own life and I remember reading she was six foot and managed to hang herself on [names ligature point]… so I went online and I was reading about it… and I went down and I tried to do it [using same ligature point] (SH1)

I thought I’ll start with [name of poison]… because I remembered Georgi Markov, the guy in the seventies that got stabbed with the umbrella… and I thought, ‘well it killed him, it will surely kill me. So, I looked up how much Georgi Markov had in his leg and it was a small pellet that went in and I thought, ‘oh that sounds good’ (SH5).

Triangulation between sources was also evident, participants cross-checking facts across sources, complementing factual knowledge about a method by seeking experiential knowledge from blogs or forum posts, or testing the feasibility of an option gleaned on one site by seeking specific details and case studies elsewhere.

As intent increased, ‘factual’ content became favoured. Self-harm patients increasingly distrusted the accuracy or authenticity of user-generated content, preferring instead a more scientific approach. Some also wished to avoid the emotional component inherent to personal accounts since this could make suicide feel ‘too real’ (SH1) and challenge their resolve. Although suicide pages were commonly used for ‘factual’ information, many self-harm patients also explored beyond these. For instance, approximately half used the general information site, ‘Wikipedia’, perceiving this to provide information not readily obtained elsewhere.

Wikipedia went, ‘It’s commonly also known as [poison], one of the six deadliest poisons in the world. If you take the inner out, that’s the poisonous bit’, and it was like, ‘oh, thanks for that’… and it said if you bake it and crush it into a powder, it’s even more deadly, so Wikipedia was just explicitly telling you how to do it. (I: Even more so than [suicide site]?) Oh yeah… Wikipedia told me the answer… they told you what was poisonous about them, how many you needed to kill yourself, how much in grams (SH5).

When I went on looking for it, I actually really wanted real information. It was ‘if I’m going to do this, I need a logical (pause), I need to know facts and Wikipedia was very good because it was very factual (SH15)

Other regularly used sources were popular medical sites such as NHS choices, patient.co.uk and WebMD (used primarily to obtain information for overdosing), and just under half consulted academic, specialist or professional resources, such as clinical case studies, pathology and toxicology reports (eg. of completed suicides), and professional medical sites.

There’s a fantastic website called [name of site]. It’s for medical (pause), I think it’s for medical training basically, not for trying to find out how to kill yourself… it’s very thorough with medical things, it has like scenarios and I found one for [drug] and I found out that anything over a gram is considered a potentially lethal dose (SH9)

[I read] quite a few medical sites, so, some of it was doctors who’d seen stories of people who had tried to kill themselves, and toxicology reports and stuff (SH6)

This was also a way of accessing information on novel methods not covered on suicide sites:

It was quite hard to get information on was how to kill yourself by injection, like injecting insulin and things like that… so the only way you could get that information was by going onto sites that were medical (SH16)

Such examples were not evident in the young people sample.

### Help-related internet use

Approximately three-quarters of the young people stated they had looked for help sites, forums, support groups and information–some by entering direct search terms such as ‘suicide help’ or the name of a help source, e.g. ‘Samaritans’. Two used the internet for this purpose only, actively avoiding suicide content. Help-related use was commonly framed in terms of looking for self-help techniques to deal with depression, information about diagnosis and treatment, and opportunities to express feelings in a safe space. Additionally, during general browsing around suicide, several came across inspirational quotes or recovery stories, which they experienced as optimistic. Internet use itself was also a common coping strategy adopted in response to feeling low. Over half described chatting on social media, participating in online dating, gaming or interest groups, and accessing entertainment (e.g. music and films) to distract themselves from negative feelings.

While help-seeking was an initial purpose for suicide-related use among many of the young people, in practice, this purpose was or could become unclear, and not always maintained once online and following links:

I used the internet for help. Um. I say for help–it’s not really ‘help’ help… I would go on the computer and look at chatrooms designed for depression. And there was one which I used to go on which was designed for people who had suicidal thoughts … it would just be full of people who were saying ‘I want to kill myself’ and of course I was one of them (YP3)

The first few days I was like ‘oh my god, this is amazing… there’s so much information that I can actually start to may be look at why I’m [self-harming] and sort of go from there’, and then when I was reading some of the comments it was quite extreme. I still read them ‘cos I was intrigued. They were encouraging each other and recommending ways of hurting themselves (YP8).

Flitting between help content and pro-suicide content or methods information was typical, particularly as mood worsened:

Because I am so up and down… sometimes I was like [suicide] is all I can look for, just that I’m going to kill myself and then other times it was, ‘I’ll go on a self-help forum for people who struggle with depression’ (YP4)

The self-harm patients, however, rarely sought online help for suicidal thinking or crisis, although some had searched for information relating to mental health problems or life events and a small, less severe group used the internet for distraction. Only one participant in this group (SH17) reported deliberately entering a suicide help search term (‘suicide help me’)–who, differing from the others, had not attempted suicide and reported no online methods research. Some had looked at suicide help content they came across while online, but usually briefly, alongside pro-suicide content, and when feeling less ill.

I did click on some [help sites] out of curiosity to see what they’d say… I’d kind of flit between looking at that out of curiosity but then going back trying to find ways of killing myself (SH16)

When I’m feeling low I don’t tend to look at [help-sites]. I tend to look at them when I’m not feeling low… [when you are feeling low] you look at what you can do to not help yourself as in look at the websites for overdoses. (SH3)

This did not occur when suicidal thoughts were intense:

When my head’s been bad, I’ve wanted to be dead and not helped, so I’m more likely to Google what can kill me quickest than how can I get help (SH9)

Many described actively avoiding or ‘blocking out’ (SH20) online help at such times, including pop-ups and support links, and ‘sifting through’ (SH2) user generated data to filter out support-giving responses. These participants explained they had decided on a course of action (suicide) and were online to research how to action it. They were single-minded in their purpose and saw ‘help’ as unwanted interference, which could make them waiver.

When I’m feeling in that state where I am sort of hunting around the internet, I am only ever looking for [suicide] method really and it’s not something that wants to be talked out of because this is the direction I’m going in and then the direction [help] would propose is going in completely the opposite direction (SH2)

Differences between the two samples in their receptivity to help online were highlighted where they encountered ‘trick sites’, which although appearing to be ‘pro-suicide’ were in fact intended to prevent suicide:

The first site that I clicked on had a misleading title but when you read it, it was someone sincerely asking you not to do it and I thought that was really nice… it was a bit of an eye opener (Int: And that was quite a helpful thing?) Yes. I think that time I did actually just stop. I just thought that’s actually quite nice and kind of put my phone down and went to sleep. (YP6)

They say they’ve got a top ten list [of methods] and then you click on it and it really is trying to give you support and I’m, ‘no, no, no. I am actually wanting to research this. I don’t want someone to help me’… it wasn’t about trying to find help or support–it was about ‘I want facts, I want statistics, I want success rates’ (SH15)

### Online communication about suicide

As with help-related use, communicating about suicide online was common amongst the young people sample but not the self-harm patients. More than half the young adults used blogging, forums, BAWW threads, discussion sites or other social media to express their feelings, manage loneliness, or engage in interactive dialogue with others. For some, this was a cathartic, one-way expression while others sought to ‘connect’ with ‘people on the same wavelength’ (YP8).

The first thing I wrote on there was ‘I want to kill myself’ and I had three people come back to me straightaway… and then I started saying how horrible I felt … I must admit, I did feel a lot better afterwards so I started using it more frequently because I knew the people on there. I felt like they were trustworthy, they gave the impression they were listening to me… some of them had similar stories, so it was nice to talk to them as well (YP3)

However, communication also assumed mixed or changeable purpose, since camaraderie could embrace the giving and receiving of support and espousing reinforcing attitudes about suicide and sharing information about methods:

I remember writing this huge post about how suicide wasn’t really selfish and this one girl who had a lot of followers re-posted it… and a lot of people liked it on hers and I was just like ‘oh wow! People get it, people understand me!’ (YP1).

I created an anonymous Instagram page. At first it was captions and quotes and stuff that I’d find and I thought were quite good… then once I saw how many people were looking at the page I started posting pictures of [self-harm] and getting more and more followers and it became addictive. It eventually got shut down… it became pro-self-harm. (YP8)

In contrast, most self-harm patients avoided generating online dialogue, again describing a wish to steer away from others’ influences. They were instead observers; almost all viewed others’ posts about methods on chatrooms, forums, and Question and Answer sites, using these as a data source that could be searched to gain insight into the experience of using methods, or to decide details of implementation (e.g. dosage) based on others experience.

All I was really looking for was to find answers to find out how much of this product it would take to kill me. That was the only point of me going on [forum]. I was never really looking to make friends and try and talk to other people… if I’m trying to kill myself I’m not looking for more friends… I wasn’t going to sign up and start writing comments and replying to people. I just wanted the answer and once I found that, I was off (SH9).

Only a quarter of self-harm patients recalled ever expressing feelings on social media or through forums, and mostly described this as a ‘one-off’ act with unclear motive:

If I’d had those conversations [in forums] earlier on, I think that would have been help and support but… I already knew that I wasn’t going to listen… I think [post] was just my way of letting it all out before [suicide] was potentially done. I knew that if I killed myself someone would go onto my laptop and check my history… and they’d be able to read my post. May be a very modern way of leaving a note (SH15)

One reported interacting extensively in pro-suicide chatrooms to seek methods advice and encouragement to act (on one occasion joining a virtual suicide pact), but was distinguished from all other participants by the persistence of her suicidal thoughts and lethality of her attempts.

I’d go back [post attempt] into the chatrooms and see if anybody had come up with any other solutions, like the other week I went in there and said, “has anybody taken a [drug] overdose?” And somebody came back and said, “Well I’ve taken forty. Although I was ill, I didn’t die, so I guess you are going to need to take more”… Others’ll go “the minimum dose is so many milligrams. What strength are your tablets? That guy may have taken forty at a lower dose”. They get quite technical with helping you to work out what you need to take to end your life… I’ll stay with it until I’m about to take the tablets and they go, ‘yeah, go for it’… You are looking for people to agree with you, that you are worthless and it is worth taking your life (SH5).

Other exceptions were explicable. For instance, echoing the self-harm patients, one young person participant had avoided communication during an episode leading up to a suicide attempt being ‘worried that someone would talk me out of it [suicide] or into it’ (YP4); while a self-harm patient had used forums extensively to seek support but primarily in relation to a mental health diagnoses, and she noted ceasing communicating during periods of acute suicidal distress.

## Discussion

This study has highlighted the variability of suicide-related internet use at differing stages of the suicidal pathway. Differences were observed between samples of young people in the community and self-harm patients admitted to hospital in relation to their purposes of use, searching and navigation, the primary content viewed, and their inclination to use the internet as a resource for help or communication. The nature of use varied according to the severity of suicidal feelings within and across samples.

Amongst the young people sample, who were mostly ambivalent about suicide and reported less suicidal behaviour, use was characterized by disorganized, speculative browsing of suicide content, largely through entering broad search terms and following links. This was mostly without clearly defined purpose other than trying to ‘make sense’ of feelings and finding common experiences to relate to. A range of content was ‘stumbled upon’, including information about methods, with the risk of being drawn further than they intended. This contrasted with the hospital sample, a higher severity group usually actively engaged in suicidal behaviour, who went online to ‘research’ methods with the intention of planning an effective attempt. Their searching was focused with triangulation between sources, and clear choices about the types of content viewed. In addition to pro-suicide content, this included consulting medical, academic and other ‘factual’ resources. Accessing help content, and expressing feelings online occurred with some purpose in the young people, but rarely amongst the self-harm patients, who were more purposeful in avoiding this. Analysis of exceptional cases in each sample were consistent with the interpretation that the severity of suicidal feelings shapes the nature of use. This was also observable across individuals’ narratives, the nature of internet use changing in line with severity as they progressed (or reversed) along a pathway to suicide.

Individuals learning about suicide methods online has been reported for some time in single case reports, the popular press, and academic publications[6, 7]. However, the processes and search strategies involved, including the recourse to ‘non-suicide’, factual material has not been detailed elsewhere. The findings reinforce the damaging impact of media portrayal of methods and of celebrity suicides in increasing accessibility of suicide and generating contagion[24]. Age differences between the two samples may have contributed to the observed differences in internet behaviour, for instance that the young people sample were more sociable online. However, 35% of self-harm patients were also aged 16–24 years.

### Strengths and weaknesses

To our knowledge, this is the first study to provide in-depth qualitative evidence from samples of suicide-related internet users drawn from the general population. Similar studies (eg.[17, 19]) have been conducted within the confines of an online survey rather than in a face-to-face context where enhanced rapport can be established and in turn, richness of response. This study therefore presents unique insights into suicide-related internet behaviour.

Recruitment in studies of self-harm and suicide is known to be difficult and to yield low response rates[25]. In this study, a range of participants from across two different populations were included and recruitment continued until consistent themes were reported and findings from ‘negative’ cases could be incorporated. This included both current suicide-related internet users and also retrospective cases, who could offer a considered reflective viewpoint[26] on how their internet use had changed over time and resolved alongside shifting experiences of distress. This increased the richness of our data. However, full diversity was not achieved since purposive sampling was limited to sex, age and severity/ suicidal intent. Clinician gatekeeping and motivation to recruit also impacted on the patient sample. Further research is needed to assess our findings in more diverse samples. The study included some who had not attempted suicide and others who had attempted but not completed suicide—though some had made serious attempts. Their internet use may differ in important ways from users who die by suicide. The individual appearing to describe the most suicidal intent, reported differing use to other participants having participated extensively in chatrooms seeking encouragement, methods advice, and on one occasion joining a virtual suicide pact.

Typical of other studies[27, 28], only a low proportion of those in the young people sample who were invited to interview agreed to participate, though this in part reflects the over-sampling of ‘hard-to-reach’ groups, such as young males as part of a purposive sampling strategy. The number of individuals in each sample was relatively small, however this was offset by the depth and extensiveness of individuals accounts. Many of those included provided rich, longitudinal narratives covering multiple episodes of suicide-related internet use at differing points along the suicidal pathway. This in turn presented multiple episodes for analysis.

While this study may not have represented the full diversity of suicide-related internet use amongst those with suicidal intent, it has identified avenues for further research and implementation.

### Implications and further research

Our findings underline the considerable potential for suicide-related internet use to increase the accessibility of suicide for those who are vulnerable. Young people with unclear suicidal intent ‘stumbled across’ pro-suicide content, which could displace intentions to find help online. Participants with severe suicidal thoughts actively used the internet to research an effective method, and often found clear suggestions. Frequently, this followed an ‘unsuccessful’ attempt—the internet becoming a resource of those already engaged in suicidal behaviour, consulted to ‘perfect’ suicide. Some purchased materials for an attempt online.

There are implications for a range of stakeholders. Awareness of how young people experiencing low mood may use the internet as part of their illness behaviour, and the inherent risks associated with this, will be beneficial for GPs, youth workers, teachers and parents, enabling them to provide additional support. Similarly, clinicians such as psychiatric liaison staff and mental health nurses, would benefit from training to increase their awareness of the nature of suicide-related internet use within the lives of those who are suicidal and self-harm, including how unexpected sources such as popular medical sites can be used in harmful ways so these are not recommended to high-risk patients. Findings show patterns of internet use reflect the status of an individual’s suicidal thinking and that particular characteristics of online behaviour may indicate severity, for instance increased specificity of searching, avoidance of online help, and recourse to ‘factual’ information sources. Inquiring about suicide-related internet use during, for example, risk assessment could support clinical decision-making by indicating ‘at risk’ patients with high suicidal intent and place clinicians in a unique position to intervene with harmful online behaviour, though this would entail training and resource implications.

Our findings also demonstrate a need for internet providers and policy makers to continue efforts to make the online world safer. Possible actions include developing existing online safety policies and expanding media guidelines[29] around the reporting of suicide to provide best practice advice for online content providers, reviewers and moderators. Restricting access to online spaces primarily existing to promote suicide would be beneficial, however findings indicate that a sole focus on pro-suicide content is misplaced since the participants with higher suicidal intent also drew heavily on ‘neutral’/ ‘factual’ content (eg. Wikipedia), professional sources of information (such as medical reports), and other ‘non-suicide’ content not intended to inform about suicide. Means of encouraging self-regulation amongst these providers should be considered, and of how to reduce risks associated with medical sites and open access research publishing, such as increased use of registration details.

Some participants reported beneficial outcomes of internet engagement such as use of help content, peer support, and the potential for information to deter participants from using some methods. These merit further examination. The interruption of suicidal thoughts by deterrence, for example, may be a useful strategy for use with severe users who often appear impervious to online help; though such an approach could also entail risk and so requires careful research. The fact many participants did look for, or at, online help content indicates a good potential for suicide prevention, though timing may be an important consideration. Online help-seeking tended to occur when suicidal intent was low, but was actively avoided at times of crisis. This provides a rationale to target individuals early in their suicide trajectory, establishing enduring support systems and help-seeking as an online behaviour that can be carried forward should distress progress. However, there is also an urgent need to develop approaches to engage individuals at imminent risk. This may include the use of well-crafted lived-experience accounts of those who have overcome suicidal crisis, which appear as relatable stories rather than explicit ‘help’ resources.

This paper has focused on the purposes and methods of suicide-related internet use. Further analyses are underway exploring how individuals processed the content gathered with respect to the meanings derived and the outcomes on their suicidal behaviour. Analysis with additional samples will examine further the assertion that suicide-related internet use can be mapped along the suicide pathway, its nature varying according to severity and intent. Psychological autopsy study may enable exploration of possible differences in use amongst those who complete suicide.

## Supporting information

S1 Text Interview topic guide. (DOCX)
