# Supplementary material for: Using the internet for suicide-related purposes: Contrasting findings from young people in the community and self-harm patients admitted to hospital
Source: PLoS One. 2018 May 24;13(5):e0197712. doi: 10.1371/journal.pone.0197712 (PMC5993121; doi:10.1371/journal.pone.0197712)
Supplement: S1 Text — (DOCX) [file pone.0197712.s001.docx]

**S1 Text: Interview Topic Guide**

***Introduction:*** *By way of introduction, could you tell me a bit about yourself?*

***Narrative of self-harm/suicide behaviour***

*When you responded to our invitation, you indicated there have been times when you’ve had thoughts about suicide/harming yourself. Can you tell me a bit about that?*

Context (family/living situation; perceived support; contact with help services)

Events leading up to self-harm/ attempt(s)

Degree of premeditation

Method used

Intent (what did want to happen as a result of harming yourself?)

***Internet use and purpose***

*You also indicated that you had seen material about self-harm/suicide on the internet.*

What is your general online behaviour? (How often, what for, devices used)

*Purpose: Can you tell me a bit more about going on online for self-harm/suicide?*

When did you first go online?

Was this accidental (e.g. online anyway), or on purpose? What was your intention?

What made you think of doing this? Why?

*Use: What did you view?*

When did you access this? (times, frequency, circumstances)

How did you access/navigate? (search strategies: terms, search engines; links, favourites)

Did you participate/ generate any material/ observe?

*Use over time: Have there been other times you’ve seen things /used the internet in this way?*

Has this changed in any way over time? (e.g. sites viewed, participation, purpose, frequency)

***Material viewed***

*I’d like to focus for a little while on some of the things you actually saw or read on the web. You mentioned you’d been to x site. Could you describe what you remember about this?*

Did you find what you were looking?

What were your thoughts/feelings about what you read?

What was it about the content that appealed or didn’t appeal to you? Was useful/ not useful

Did you choose between content? How? What drew you?

What impact did it have on you? (Behaviour/ intentions/ emotional feelings)

What messages did you take away?

Have you gone back since?

***Material generated***

Now could you describe to me any material you generated? (content; where posted; purpose; responses received; interactive exchange).

***Impact of internet on behaviour and feelings***

*Thinking back to the time(s) you had thoughts about harming yourself/harmed yourself, tell me about the role the internet played for you?*

How important do you think the Internet was to what happened? In what ways?

Negative (e.g. choice of method/ use of method/ encouragement)

Positive (e.g. dissuading/someone to talk to/relief of feelings/self-help/direct to help)

How might things be different if you hadn’t gone online?

Did the internet give you anything unique? (that you couldn’t get elsewhere)

Over time/thinking more generally, what impact would you say has the internet had on:

Your thoughts and feelings about self-harm

The way you cope with feelings about suicide/self-harm?

What other things have influenced you? Are these more, or less important than the internet?

***General views on Internet as a source of harm/ support***

Is the Internet harmful for people when they are feeling suicidal/like self-harming?

Can the Internet be positive/helpful – for you/ others? How?

What is ‘bad’/ ‘good’ material?

How could harm be lessened?

***Evaluation of existing support materials/approaches***
